# Questions of the Day
*Questions of the Day. By the Creature of the Hour.


**Published:** 1857-10

**Authors:** 


### QUESTIONS OF THE DAY.*

*Questions of the Day* are by an anonymous writer. They are a series of elegantly written, simple, soul-earnest essays. Those on our "Criminal Population"; "Rich and Poor"; "Sanitary Reform"; "Sewage and Agriculture"; "Sports for the People"; interest us most deeply. Speaking of the erection of churches *versus* the erection of comfortable dwellings for the poor, the author thus remarks in his *Sanitary Reform* essay.

"It is vain to taunt us with the zeal and self-denial of our forefathers, in erecting abbeys and cathedrals as a tribute to the glory of Him who made them. The enchanting beauty of the steepled minster is not denied. When we look up at the lofty proportions of such a building let us see it as it is seen by those who look down on it from above....And let us sympathize with such survey from a higher sphere, as it lights upon some hovel darkened by the shadow of those towers, low, close, dreary, damp, and filthy, acknowledged to be the adverse to the maintenance of sound health, cheerfulness, and decency, and yet accounted by minster builders quite good enough for those to dwell in, who are made in the likeness of their maker. These, and such as these, who live and die by millions, uncared for in the midst of evil physical and moral, evil whereof much is certainly preventible, these are they of whom ought to be made the living stones of the living temple. What can we do better with our substance than help in fitting them to occupy the place of so high a calling in the order of the new creation?"

There is poetry of thought in these sentiments to which many hearts will respond. Throughout the work the same generous feeling flows; and although we may not agree with the author in all he says, we thank him earnestly for his earnest offering, of the which, whoever reads, will be wiser and better for the effort.

### WORK, FRESH AIR, AND EXERCISE.†

The Torquay Early Closing Association have an earnest and able associate in Dr. Radclyffe Hall. He gives in this lecture

* Questions of the Day. By the Creature of the Hour.

